# Visual Rehabilitation by Scleral Fixation of Posterior Chamber Intraocular Lenses in Amblyopic Aphakic Children

**DOI:** 10.4103/0974-9233.51994

**Published:** 2008

**Authors:** Anuradha Ganesh, Alexander A. Bialasiewicz, Sana M. Al-Zuhaibi, Buthaina I. Sabt, Shyam S. Ganguly

**Affiliations:** 1 From the Department of Ophthalmology and School of Ophthalmic Technicians, Sultan Qaboos University College of Medicine and Health Sciences, Muscat, Oman; 2 From the Magrabi Eye and Ear Center, Al Ahli Specialty Hospital, Doha, Qatar; 3 From the Department of Epidemiology and Medical Statistics, Sultan Qaboos University College of Medicine and Health Sciences, Muscat, Oman

**Keywords:** cataract, trauma, pediatric IOL, amblyopia, scleral fixation

## Abstract

**Background/Aims:**

To report on the outcome of scleral fixated posterior chamber intraocular lens (S-IOL) implantation in aphakic amblyopic children after 1 year.

**Methods:**

Amblyopic children with aphakia after traumatic and congenital cataract surgery unsuitable for spectacle or contact lens correction were operated with an anterior vitrectomy and inside-out double thread scleral fixation of an Alcon CZ70BD pcIOL. Refraction and vision was compared after 12 months.

**Results:**

From 2001-2006, 23 S-IOLs were implanted in 16 children (19 unilateral, 4 bilateral) aged 2-16 years: 10 eyes with traumatic [Group A], and 13 eyes with congenital cataracts including 3 eyes with ectopia lentis [Group B]. Preoperative UCVA compared to postoperative UCVA improved in 9/10 eyes in group A and 12/13 eyes in group B. Preoperative BCVA compared to postoperative UCVA improved in 9/10 eyes (90 percent) in group A and 4/13 eyes (31 percent) in group B. Mean age at surgery in group A was 6.8 years (1.5-16yrs) and in group B 10.5 years (4-16 years). More than one year elapsed in 2/10 eyes of group A and 8/13 eyes in group B. Postoperative refraction was within 2.0D of target in 17/23 eyes. Complications included temporary IOP rise in 2, vitreous hemorrhage in 1, and iris capture in 3 eyes. Two eyes required revision surgery.

**Conclusion:**

S-IOL implantation may be beneficial for aphakic children lacking other means for visual rehabilitation to improve vision. Amblyopia may be improved in most trauma, but only few congenital cataract eyes.

Visual rehabilitation of aphakic children in many developing countries by spectacles and contact lenses is difficult due to the prevailing environment, culture and socioeconomics. Because of the noncompliance of patients and parents amblyopia is commonplace in Oman.^1^,^2^

Recent studies have shown that the implantation of scleral fixated posterior chamber intraocular lenses (S-IOL) is feasible and renders more favorable results in children over 2 years of age if non-compliant with spectacles or contact lenses.^3^–^9^ Implantation of IOLs in children less than 2 years is still controversially discussed.^10^,^11^ Therefore, we have performed a study to compare the outcomes of secondary intraocular lens implantation in aphakic eyes of children older than 2 years previously operated for traumatic and congenital cataracts.

## Patients and Methods

### Patients:

From 2002-2005, 28 patients under 16 years of age were operated in a single center study at the Sultan Qaboos University Hospital in Muscat, Oman. 23 eyes of 19 patients were examined one year after S-IOL surgery and were eligible for this study.

### Indications:

A secondary IOL implantation was considered in all children with unilateral aphakia without capsular support, because contact lens correction in Oman has been shown unsuccessful and costly, and in children with bilateral aphakia without capsular support who showed poor compliance with spectacles and contact lenses.^2^ Iris fixated and other anterior chamber IOLs and corneal refractive interventions were not considered. Written informed consent was obtained from the parents in all cases.

### Examinations:

All patients underwent a detailed ophthalmic examination that included preoperative and postoperative uncorrected (UCVA) and best corrected (BCVA) visual acuity, orthoptic evaluation, slit-lamp examination of the anterior segment, and intraocular pressure. The pcIOL power was calculated using the SRK II formula by A-scan biometric (OcuScan^®^RxP, Alcon^®^), topographic (ATLAS^®^, Zeiss-Humphrey) and keratometric (NIDEK) readings. IOL power selections were modified according to the charts by age.^12^

### Surgery:

Under general anesthesia transconjunctival scleral troughs were made at 2 and 8 o'clock 2 mm behind the limbus, and the anterior chamber was entered through a scleral tunnel. Anterior vitrectomy was performed prior to pcIOL (Alcon CZ70BD) implantation in all cases. The pcIOL model used in this study was A double-armed 10×0 polypropylene (Ethicon, Inc.) suture on a bent or straight needle was used to secure the pcIOL (with a first knot on the haptic) in the sulcus by perforating the sclera in the lamellar troughs at 2 and 8 o'clock. In that way, 2 threads were holding the IOL on each side. The scleral suture was then tied to itself and the sclera, and the knot was buried in the trough. Thjs maneuver was performed to prevent possible IOL subluxation due to breakage of one thread in later years as has been previously reported.^7^ A subconjunctival injection of 2mg dexamethasone phosphate and 20mg gentamicin sulfate was given at the end of surgery.

### Postoperative Management:

Prednisolone acetate eye drops (1 percent) were administered hourly for the the first 24 hours and then every 2 hours, and gentamicin 0.3 percent drops every 6 hours. The frequency of the steroid drops was tapered according to the degree of inflammation. Amblyopia was treated by occlusion therapy and/or daily atropine instillation in the good eye.

### Statistics:

Data were evaluated by descriptive statistical tests (Wilcoxon's and paired t-test). Comparison of means was carried out using the Kruskal Wallis test. Pearson's coefficients were calculated to study the association between two variables.

## Results

S-IOLs were implanted in 10 eyes with aphakia after surgery for traumatic cataracts [Group A], in 10 eyes with congenital cataracts and in 3 eyes with ectopic lenses [Group B]. Patients in Group B were slightly older at the time of S-IOL implantation (Group A: mean age 7.3 years th ± 3.6; Group B: mean age 10.38 years th ± 3.77). Primary S-IOL implantation during lensectomy was done in 3 eyes with ectopic lenses due to Weill-Marchesani and Usher syndrome. Secondary IOL implantation was performed in all other eyes. (see Tables 1,2)

**Table 1:** Outcome of Secondary S-IOL Implantation in Amblyopic Aphakia after Surgery for Congenital Cataracts and Lens Dislocation

No. Eye	MRN	DOB	Etiology	Age at 1st Surgery	Age at S-IOL Surgery	Delay	UCVA Pre S-IOL*	BCVA Pre S-IOL*	UCVA 1y post OP	Target Refraction	Post OP Refraction	Secondary Findings
1	130440	12/07/95	congenital cataract	3.5y	4y (secondary)	6m	0.001	0.001	0.65	emmetropia	−1.0/−1.25/−120°	-

2	69279	01/01/94	congenital cataract	8y	8yrs (secondary)	4m	0.001	0.05	0.15	−0.5	−2.5/−3.0/9-0°	-

3	73766	17/05/95	congenital cataract	7m	7y (secondary)	6m	0.001	0.3	0.3	+1.0	+2.0/−1.75/−120°

4	73766	17/05/95	congenital cataract	7m	7y (secondary)	6m	0.001	0.3	0.3	+1.0	+2.0/−1.0/1-70°	-

5	22303	01/03/90	congenital cataract	3y	11y (secondary)	8yrs	0.001	0.001	0.001	emmetropia	−1.0/−1.0/1-20°	-

6	70518	19/06/94	congenital cataract	1y	8y (secondary)	7yrs	0.001	0.65	0.65	emmetropia	+0.5/−2.5/1-55°	-

7	134150	01/09/87	congenital cataract	1y	16y (secondary)	15yrs	0.001	0.2	0.2	+0.5	+0.5/−0.75/−90°	-

8	134150	01/09/87	congenital cataract	1y	16y (secondary)	15yrs	0.001	0.2	0.2	+0.5	+0.75/−1.0/−80°	-

9	134148	01/09/89	congenital cataract	1y	14y (secondary)	13yrs	0.001	0.5	0.5	emmetropia	−1.5/130°	-

10	134148	01/09/89	congenital cataract	1y	14y (secondary)	13yrs	0.001	0.4	0.4	emmetropia	−1.75/65°	-

11	185717	01/03/96	lens dislocation	8y (diagnosis)	9y (primary)	1yr	0.001	0.075	0.5	−0.25	+0.5/−1.0/1-60°	Weill Marchesani syndrome

12	185717	01/03/96	lens dislocation	8y (diagnosis)	9y (primary)	1 yr	0.001	0.3	0.3	emmetropia	−3.0/−0./11-0°	Weill Marchesani syndrome

13	242590	01/05/93	lens dislocation	11y (diagnosis)	12y (primary)	1yr	0.001	0.075	0.15	emmetropia	−1.5/−1.5/1-50°	retinal dystrophy, Usher syndrome

* Low vision decimalized according to Holladay TA: Visual acuity measurements. JCRS 30/2(2004)

**Table 2:** Outcome of Secondary S-IOL Implantation in Amblyopic Aphakia after Surgery for Traumatic Cataracts

No. Eye	MRN	DOB	Etiology	Age at 1st Surgery	Age at S-IOL Surgery	Delay	UCVA Pre S-IOL*	BCVA Pre S-IOL*	UCVA 1y postOP	Target Refraction	Post OP Refraction	Secondary Findings
1	219847	10.11.02	Traumatic cataract	1.5y	2y (primary)	6m	0.0001	0.0001	0.01	emmetropia	−5.0/−2.0/1-60°	-

2	251754	01/01/02	Traumatic cataract	3y	5y (secondary)	2yrs	0.001	0.05	0.05	emmetropia	+3.75/−0.5/−141°	corneal scar

3	223389	01/01/98	Traumatic cataract	6y	6y (secondary)	2m	0.001	0.2	0.65	+0.75	+3.0/−4.0/1-40°	-

4	226022	01/09/98	Traumatic cataract	6y	6y (secondary)	2m	0.0001	0.0001	0.4	+1.0	+1.5/−2.0/1-45°	-

5	225901	01/09/98	Traumatic cataract	6y	6y (secondary)	7m	0.001	0.001	0.05	emmetropia	−0.25/−5.0/−135°	keratoconus

6	216392	01/05/97	Traumatic cataract	7y	7.5y (secondary)	6m	0.001	0.001	0.01	+0.75	−1.5/−8.75/−160°	keratoconus

7	193651	01.07.96	Traumatic cataract	7y	7y (secondary)	8m	0.001	0.001	0.001	emmetropia	unassessable	corneal scar

8	220801	01/02/97	Traumatic cataract	7y	7.5y (secondary)	6m	0.001	0.5	1.0	emmetropia	+0.5/−3.0/2-0°	-

9	119773	08/08/92	Traumatic cataract	10y	10y (secondary)	2m	0.001	0.001	0.8	emmetropia	−0.25/−2.0/−120°	-

10	70905	01.06.85	Traumatic cataract	10y	16 (secondary)	6yrs	0.001	0.001	0.9	emmetropia	+0.75/−2.0/−121°	-

* Low vision decimalized according to Holladay TA: Visual acuity measurements. JCRS 30/2(2004)

### Unilateral / Bilateral Surgery:

The major reason for S-IOL implantation was unilateral aphakia after cataract surgery for trauma (10 eyes) and congenital cataract (5 eyes). Four patients (3 congenital cataracts, 1 ectopic lens) had a bilateral S-IOL implantation.

### Postoperative Vision:

Because best postoperative UCVA was the target of surgery, and patients were operated because they did not comply with visual aids, postoperative BCVA was not included in this evaluation of postoperative vision. Preoperative UCVA vs postoperative UCVA improved in all 23/23 eyes significantly from mostly finger counting to a 5 meter vision (p=0.001).

Preoperative BCVA vs. postoperative UCVA improved in 8/10 eyes with trauma, but only in 4/13 eyes with congenital cataracts and ectopic lenses (p=0.03).

Compliance with amblyopia therapy was difficult in all patients. Persistent amblyopia after one year of treatment was seen in 11/23 eyes (48 percent).

### Postoperative Refraction after 1 Year:

Postoperative refraction was within th ± 2.0 D of the estimated spherical equivalent in most of the eyes. One eye was miscalculated with a refraction of -5.0/-2.0/160°, and 5/23 eyes (4 trauma, 1 congenital cataract) had a refraction of 3D different from target.

### Amblyopia:

Prior to the S-IOL implantation, deprivation amblyopia was observed in all eyes. Persistence of amblyopia and limited visual rehabilitation after S-IOL surgery was a more significant problem in the congenital cataract compared to the trauma group (p=0.012) (Tables 1,2). Other factors that precluded visual recovery in both groups were retinal dystrophy, corneal scars, and keratoconus.

### Delay until Refractive S-IOL Implantation:

The delay between primary lensectomy and S-IOL implantation was significantly different with a mean of 13.5 months (<2 months to 6 years) in Group A and a mean of 70 months (< 2 months to 15 years) in Group B (p=0.026).

### Postoperative Complications:

Postoperative complications included temporary elevation of intraocular pressure (2 eyes), and vitreous hemorrhage (1 eye). A pcIOL decentration was observed in 3 eyes necessitating revision surgery in 2 eyes. No retinal or infectious complications were encountered.

## Discussion

Our study shows that scleral fixated pcIOL implantation in children of a developing country with restrictive cultural beliefs about spectacles and environmental limitations of contact lens use is an effective means to correct aphakia in eyes lacking posterior capsular support.

Posterior chamber intraocular lens implantation has been accepted to offer the most satisfactory visual outcome in aphakic children above 2 years of age for over 20 years.^10^,^11^ The use of the procedure is controversially discussed for infants under the age of two years due to IOL calculation problems with regard to small globe size, axial length changes, corneal flattening and postoperative complication rate.^3^,^12^–^15^ For IOL calculations, we applied the age-adjusted corrections suggested by Crouch.^12^ We did not substract 1D from the final power of the S-IOL as had been suggested previously.^5^

In our study an improvement in visual acuity between pre- and postoperative UCVA was achieved in 21/23 eyes (91 percent), and the postoperative (=target) UCVA vs. the preoperative BCVA improved in 12/23 eyes (52 percent): 2/10 eyes (20 percent) of the congenital cataracts, 2/3 eyes (66 percent) of the ectopic lenses and in 8/10 eyes (80 percent) of the trauma group. The latter data may be interpreted as an effective postoperative amblyopia treatment, which was significant in the trauma vs. the cataract group. Only two of the 10 patients with congenital cataracts who underwent secondary pcIOL implantation experienced an improvement of amblyopia. However, a point to note is that the cohort of patients with congenital cataracts was older than the cohort with traumatic cataracts. We attributed the difference in visual performance in the congenital and traumatic cataract groups to irreversible amblyopia in the former. Previous studies have shown that the rate of refractive change in eyes with scleral fixated pcIOLs is the same as that in children with in-the-bag pcIOL implantation.^5^,^16^

Predicted postoperative refraction was on target in 17/23 eyes (74 percent), which was comparable to previous and long-term studies.^7^–^9^ However, postoperative refraction results were significantly less variable in groups B (congenital cataracts and ectopic lenses) vs. group A (trauma), probably because of the underlying corneal pathologies. Ten of 23 eyes maintained preoperative visual acuity levels.

Scleral fixated pcIOL implantation in children is technically more difficult than routine posterior chamber IOL implantation. Anterior vitrectomy as performed in our study is required in all cases to decrease the probability of vitreous traction leading to secondary retinal detachment.^17^

Complications in our study included temporarily elevated IOP unrelated to the IOL insertion, vitreous hemorrhage due to attempted posterior synechiolysis and pcIOL subluxation requiring revision surgery in 2/23 eyes (8.7 percent). This low rate may, of course, increase in the following years, a fact, which has been published recently.^7^ Erosion, breaking or wearing away of the 10×0 polypropylene thread is of some concern, since it has been shown that fibrous reactions around the IOL haptics is lacking.^18^,^19^ However, we have applied a method by which 2 threads each at 2 and 8 o'clock are holding the IOL with knots in troughs,^20^ and expect a decreased possibility of IOL subluxation. Complications encountered in our study are comparable with those seen in other pediatric scleral-fixated pcIOL studies, and retinal problems arising from the procedure or endophthalmitis due to a fistula have not been encountered.^17^,^21^ No study to date exists with long-term follow-ups into adulthood.

In conclusion, scleral fixation of pcIOLs provides an effective means to correct aphakia in eyes without posterior capsular support in patients otherwise doomed by cultural, environmental and socioeconomic reasons for deep amblyopia.
